# Cavo-atrial thrombectomy prior to hepatectomy for hepatocellular carcinoma with tumor thrombus in the right atrium: a case report

**DOI:** 10.1186/s40792-019-0620-y

**Published:** 2019-04-11

**Authors:** Shun-ichi Ariizumi, Chizuo Kikuchi, Fumiaki Tokitou, Shingo Yamashita, Yoshihito Kotera, Akiko Omori, Takaaki Kato, Satoshi Nemoto, Hiroshi Niinami, Masakazu Yamamoto

**Affiliations:** 1 Department of Surgery, Institute of Gastroenterology, Tokyo Women’s Medical University, Kawada 8-1, Shinjuku-ku, Tokyo, 162-0054 Japan; 2 Department of Cardiovascular Surgery, Tokyo Women’s Medical University, Tokyo, Japan

**Keywords:** Hepatocellular carcinoma, Tumor thrombus, Right atrium, Thrombectomy

## Abstract

**Background:**

Hepatocellular carcinoma (HCC) with tumor thrombus (TT) in the right atrium is a critical condition. The general consensus is to perform hepatectomy prior to cavo-atrial thrombectomy because of the risk of uncontrollable bleeding during the liver transection after heparinization. However, sudden cardiac arrest due to the ball-valve effect and pulmonary embolism have been reported in cases of TT. Cavo-atrial thrombectomy prior to hepatectomy for HCC with TT in the right atrium was successfully performed to prevent sudden cardiac arrest and pulmonary embolism.

**Case presentation:**

Tumor thrombectomy under cardiopulmonary bypass with heparin and electrical ventricular fibrillation prior to hepatectomy was successfully performed to prevent sudden cardiac arrest or pulmonary embolism in a 75-year-old woman with a huge HCC and TT in the right atrium. After the neutralization of heparin, right hepatectomy with tumor thrombectomy in the inferior vena cava was performed. The total operation time was 9 h, and the total blood loss was 8200 mL. The patient’s postoperative course was uneventful, and she was discharged 14 days after surgery. One year after surgery, she is alive with HCC recurrence in the lung.

**Conclusions:**

Cavo-atrial thrombectomy prior to hepatectomy for HCC with TT in the right atrium can be performed safely to prevent sudden cardiac arrest and pulmonary embolism by collaboration of cardiovascular surgeons and gastroenterological surgeons.

## Background

Hepatocellular carcinoma (HCC) with tumor thrombus (TT) in the right atrium is a critical condition [1]. There is no standard treatment strategy, and surgery is challenging. The median survival is reported to be from 1 to 4 months, and it may be complicated by lung metastasis, pulmonary embolism, heart failure, and sudden cardiac death [1, 2]. There are no effective nonsurgical treatments, and while long-term survivors have been reported after surgery, surgical mortality is reported to be 15% [1–5]. The general consensus is to perform hepatectomy prior to cavo-atrial thrombectomy, because of the risk of uncontrollable bleeding during the subsequent transection of the liver after heparinization [6]. However, cavo-atrial thrombectomy prior to hepatectomy should be considered in cases with TT which fully enters the right atrium, reaches the tricuspid valve, and is of the pedunculated type, in order to prevent the ball-valve effect or pulmonary embolism [2].

## Case presentation

**Fig. 1:**
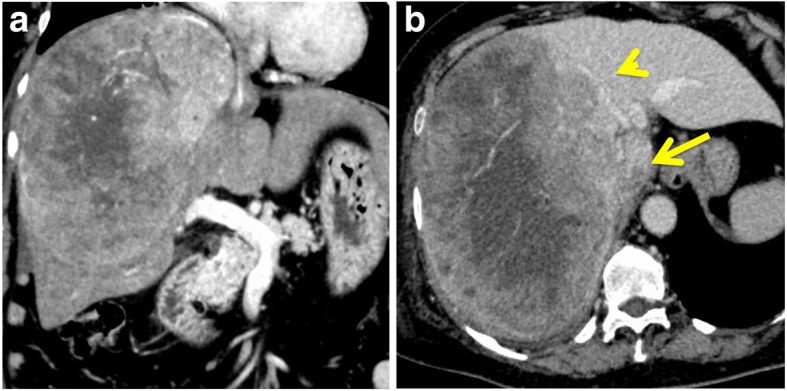
CT showed a huge tumor, 21 cm in diameter, in the right liver (**a**). The tumor compressed the middle hepatic vein (arrowhead) and inferior vena cava (arrow) (**b**)

**Fig. 2:**
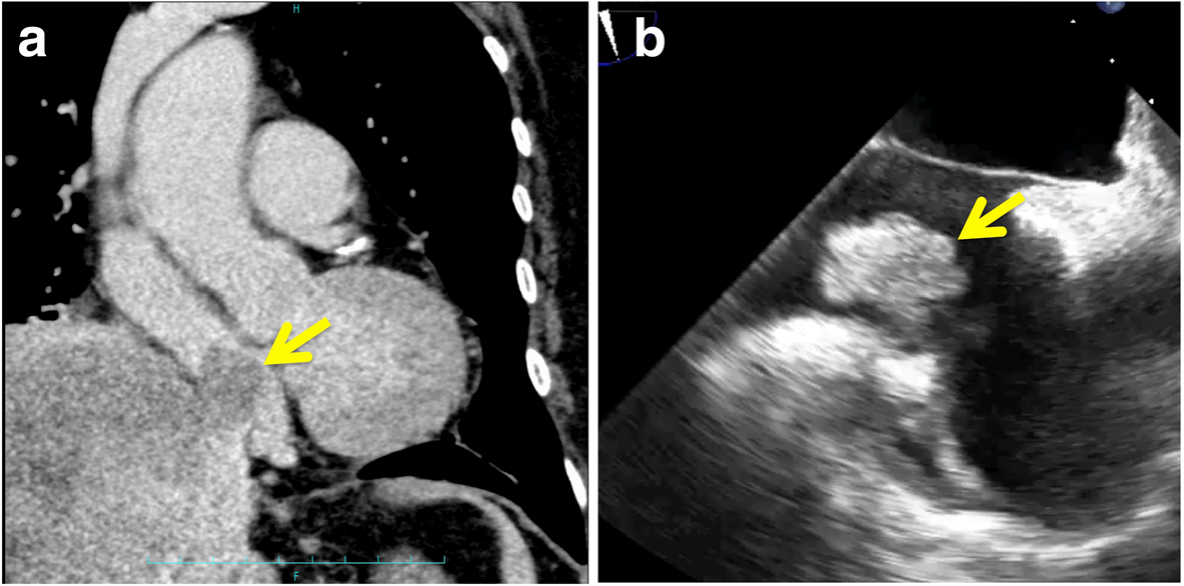
CT showed a tumor thrombus in the right atrium (**a**, arrow). The tumor thrombus moved with the heartbeat and reached the tricuspid valve on transesophageal ultrasound (arrow) (**b**)

The patient was a 75-year-old woman who presented with severe bilateral leg edema and epigastralgia. There was no past history of chronic hepatitis or blood transfusion. Admission laboratory tests revealed an elevated serum aspartate aminotransferase level (48 U/L), mildly decreased serum albumin (3.6 g/dL), mildly decreased serum prothrombin time (74%), mildly elevated D-dimer (12.9 μg/mL), and mildly elevated fibrin degradation product (12.6 μg/mL). The tumor marker AFP was slightly elevated to 585 ng/mL. The Child-Pugh classification was A, and the indocyanine green retention rate at 15 min was 12%. CT scans showed a huge tumor, 21 cm in diameter, in the right liver and TT, 37 mm in diameter, in the right atrium (Figs. 1a, b and 2a). The TT was pedunculated, swung like a pendulum with the heartbeat, and reached the tricuspid valve on a transesophageal ultrasound video (Fig. 2b). The symptomatic patient had a risk of sudden death due to TT in the right atrium. Therefore, we explained the high surgical mortality rate to the patient and her family, and they decided to proceed with surgery.

**Fig. 3:**
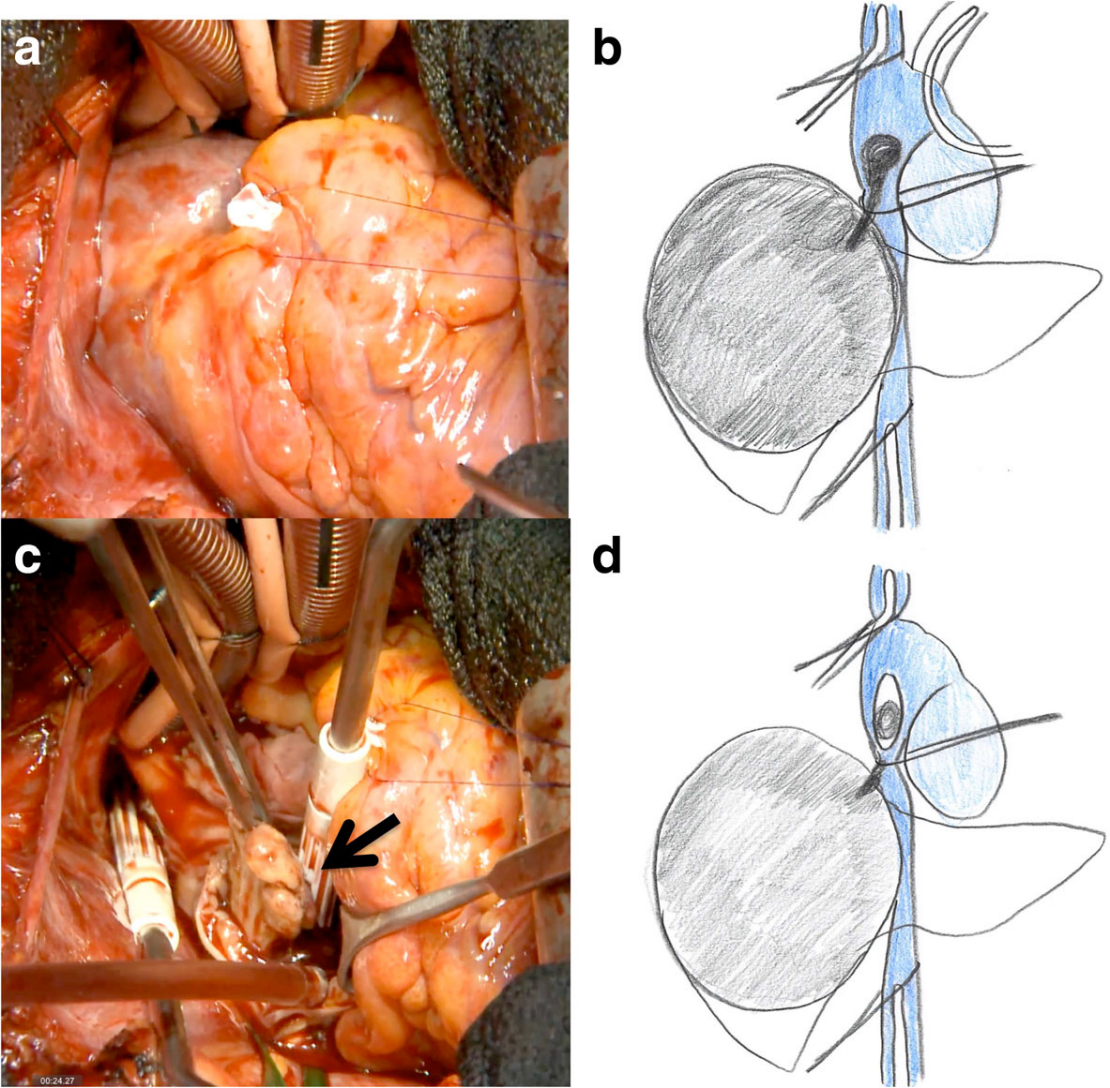
Tumor thrombectomy in the right atrium under electrical ventricular fibrillation and cardiopulmonary bypass (CPB) was performed after intravenous injection of heparin sodium (**a**, **b**). The TT in the right atrium was removed from the right atrium without pulmonary embolization (**c**, **d**). The open heart surgery time was 23 min

**Fig. 4:**
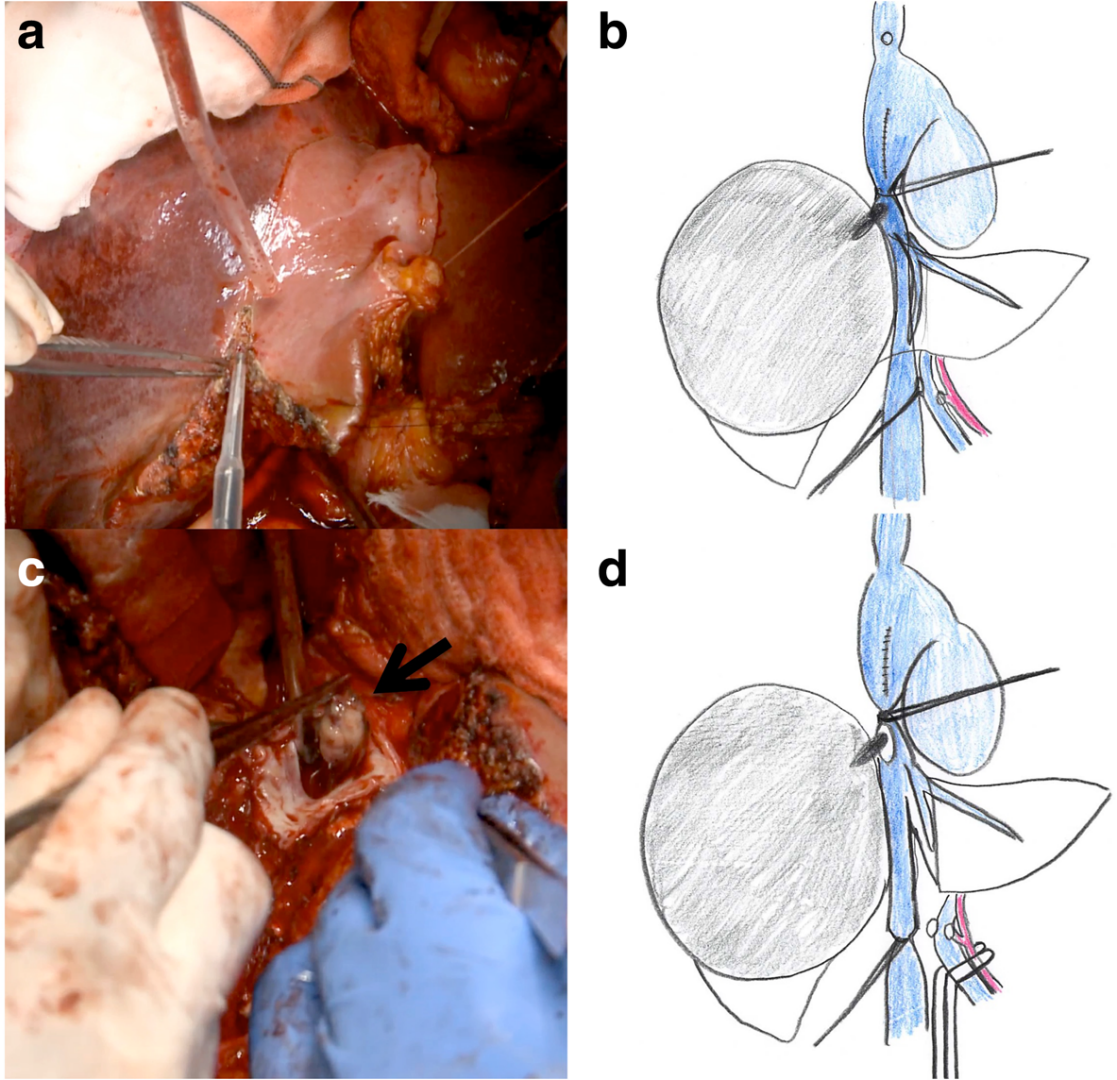
Right hepatectomy by anterior approach (**a**, **b**) and IVC tumor thrombectomy were performed under total hepatic vascular exclusion after neutralization of heparin (**c**, **d**)

**Fig. 5:**
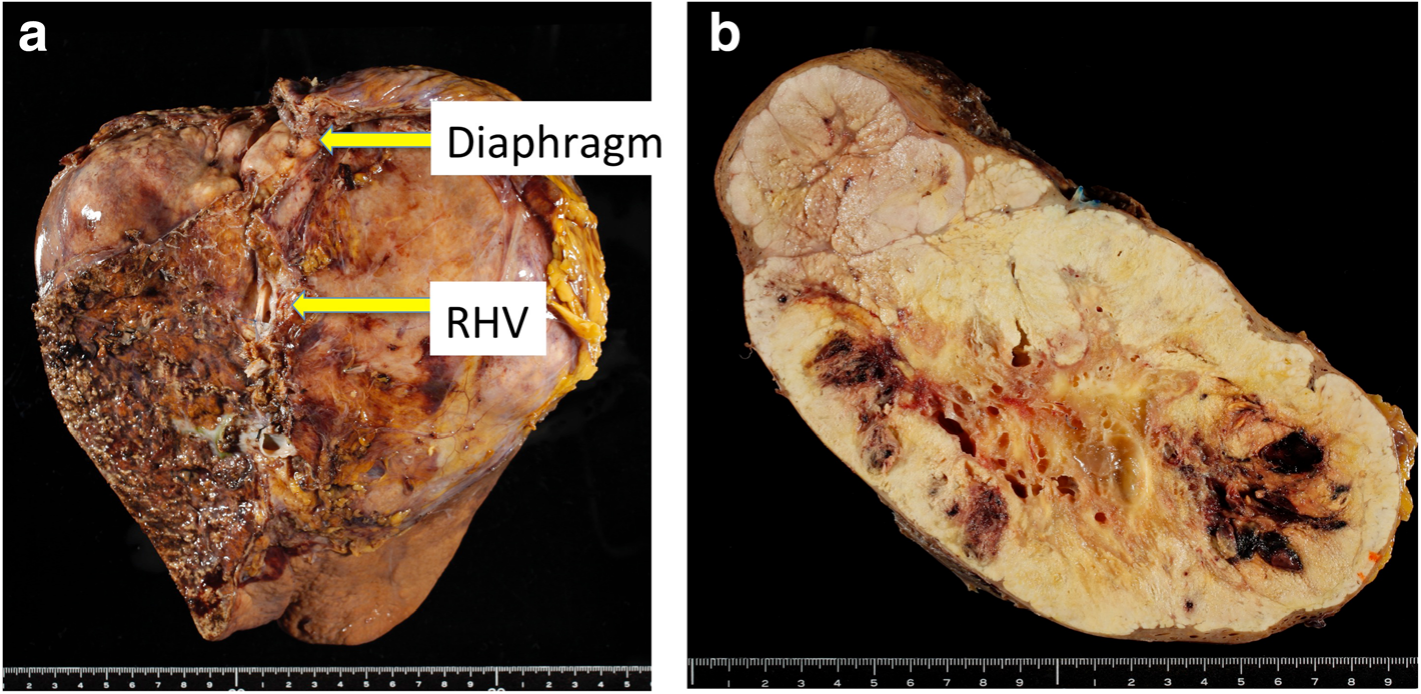
The tumor showed a massive type macroscopically and HCC invaded to the diaphragm (**a**, **b**)

At first, a partial sternotomy was made, and cardiopulmonary bypass (CPB) via the aorta, superior vena cava, and right femoral vein and electrical ventricular fibrillation were performed after intravenous injection of 25,000 units of heparin sodium. Although the TT in the right atrium was very soft and fragile, thrombectomy from the right atrium and inferior vena cava (IVC) was successful (Fig. 3a–d). After the removal of TT, the IVC was clamped with a tourniquet above the diaphragm and the right atrium was sutured with 5–0 Prolene. After DC defibrillation was carried out, CPB was stopped, the tourniquet on the IVC was released, and 100 mg protamine was administered for the neutralization of heparin. The open heart surgery time was 23 min, and the CPB time was 42 min. Next, a reverse T incision was made, and right hepatectomy by anterior approach and IVC tumor thrombectomy were performed. After ligation of the anterior and posterior Glissonean pedicles, the liver parenchyma was transected under the Pringle maneuver and IVC clamping below the liver. After the IVC and right hepatic vein were confirmed by anterior approach, the IVC was opened and a residual TT in the IVC was removed under total hepatic vascular exclusion (THVE) (Fig. 4a–d). Finally, the huge tumor was removed with the diaphragm without pulmonary embolism. The total operation time was 9 h, and the total blood loss was 8200 mL. The tumor was of the massive type macroscopically (Fig. 5), and cancer cells showed moderately to poorly differentiated HCC with invasion to the portal vein, hepatic vein, and diaphragm. The patient’s postoperative course was uneventful, and she was discharged 14 days after surgery. She is still alive 14 months after surgery with recurrence in the lung.

## Discussion

The general consensus is to perform hepatectomy prior to cavo-atrial thrombectomy, because of the risk of uncontrollable bleeding during the subsequent transection of the liver after heparinization. However, in cases of a large TT which enters the right atrium, reaches the tricuspid valve, and is of the pedunculated type, sudden cardiac arrest due to the ball-valve effect and pulmonary embolism have been reported. Therefore, cavo-atrial thrombectomy prior to hepatectomy should be considered as one of the options for HCC with TT in the right atrium [1–6].

Tsang et al. reported a case of HCC with TT in the right atrium treated with combined cavo-atrial thrombectomy and hepatectomy [2]. According to their report, liver transection by the anterior approach was carried out before thrombectomy. However, hepatectomy was converted to thrombectomy because unstable hemodynamics and uncontrollable bleeding (blood loss was 30 L) developed as a result of right ventricular inflow obstruction secondary to the ball-valve effect. In our present case, cavo-atrial thrombectomy prior to hepatectomy was performed to prevent the ball-valve effect or pulmonary embolism because the TT swung like a pendulum with the heartbeat on a transesophageal ultrasound video. Transesophageal ultrasound video of a TT is useful for deciding to perform cavo-atrial thrombectomy prior to hepatectomy.

Sakamoto and Nagano classified TT in the IVC and/or right atrium into type I–III, and the intracardiac type (type III) requires hepatectomy plus thrombectomy under CPB. There have been 14 previous reports of cases in which HCC with TT in the right atrium was successfully removed with hepatectomy prior to thrombectomy and CPB by collaboration of cardiovascular surgeons and gastroenterological surgeons (Table 1) [3, 7]. According to the reports, eight patients had no complications after surgery. However, one patient developed DIC, one patient had bleeding, and two patients had pleural effusion after surgery. Wakayama et al. reported six cases of HCC with TT in the right atrium treated with hepatectomy prior to thrombectomy with CPB, two cases had controllable complications, but there was no surgical mortality [4]. At our institute, six patients with HCC and TT in the right atrium underwent hepatectomy prior to thrombectomy with or without CPB between 1986 and 2001. Three of six patients died of sudden cardiac arrest during surgery. We therefore stopped surgery for HCC with atrial TT since 2001.

**Table 1:** Reports of cases with HCC and tumor thrombus in the right atrium

Author	Year	Age/sex	Size of HCC	Size of TT	Liver or heart surgery first	Hepatectomy	CPB or THVE	Cardiac arrest	Temperature	Heparin	Blood loss	Operation time	Complication	Survival	Recurrence
Onitsuka	1990	59/male	15	N.D.	Liver first	Left HR	CPB	Arrest	Hypothermia	N.D.	N.D.	N.D.	N.D.	7 months alive	No
Tsuzuki	1991	57/male	5	5.2	Liver first	S4	CPB	No arrest	Normal	3 mg/kg	6720	13 h 52 min	DIC	5 months dead	Liver
Fujisaki	1991	38/female	8.5	N.D.	Liver first	Left HR	CPB	Arrest	Hypothermia	N.D.	9400	14 h 54 min	No	15 months alive	No
Sano	1999	59/male	10	N.D.	Liver first	Right HR	Without CPB, THVE				14,321	8 h 20 min	No	56 months dead	Liver
Ozeki	2000	70/male	11	3	Liver first	Right HR	Without CPB, THVE				3450	9 h 10 min	No	26 months alive	No
Yokoi	2000	54/male	7	7	Liver first	Left HR	CPB	N.D.	N.D.	3000 U/kg	14,000	8 h 55 min	Bleeding	12 months alive	Liver, bone
Wu	2000	42/male	1.5	15	Liver first	Partial resection	CPB	Arrest	Hypothermia	N.D.	7000	11.2 h	No	14 months dead	Lung, bone
Yogita	2000	61/male	5	N.D.	Liver first	Left HR	CPB	No arrest	N.D.	0.3 mg/kg	2910	11 h 20 min	No	56 months dead	Lung
Miyazawa	2004	55/male	7	4	Liver first	Right HR	CPB	N.D.	N.D.	N.D.	4500	6 h 30 min	No	12 months alive	No
Sugimoto	2004	55/male	14	N.D.	Heart fiirst	Left HR	CPB	No arrest	N.D.	N.D.	3735	8 h 20 min	No	11 months alive	Liver, lung
Uemura	2004	60/male	5	N.D.	Liver first	Right HR	Without CPB, THVE				N.D.	545 min	No	14 months alive	No
Tani	2006	68/male	10	3.5	Liver first	Right HR	CPB	N.D.	N.D.	N.D.	2750	7 h 29 min	No	11 months alive	Liver
Lin	2007	57/male	4.5	N.D.	Liver first	Right HR	N.D.	N.D.	N.D.	N.D.	N.D.	N.D.	Multiple organ failure, dead	3 days dead	N.D.
Florman	2009	55/male	18	5	Liver first	Left HR	CPB	Arrest	N.D.	N.D.	N.D.	6 h	No	3 months alive	No
Leo	2010	45/male	N.D.	N.D.	Liver first	Left HR	CPB	Heart fibrillated	Hypothermia	N.D.	N.D.	N.D.	Pleural effusion	6 months alive	Lung
Kyokane	2010	64/female	2.2	5	Liver first	Right HR	Without CPB, V-V bypass				7714	11 h 35 min	No	24 months alive	Lung
Inoue	2011	72/male	5	3	Liver first	Left HR	CPB	No arrest	N.D.	7000 U	4180	13 h 25 min	No	27 months alive	No
Li	2012	64/male	5.3	N.D.	Liver first	Posterior sectionectomy	CPB	Arrest	N.D.	N.D.	11,000	405 min	No	6 months dead	Liver
		44/male	10.7	N.D.	Liver first	Left HR	CPB	No arrest	N.D.	N.D.	1200	360 min	Pleural effusion	38 months dead	Liver
Li	2013	40/male	10	N.D.	Liver first	Right HR	CPB	N.D.	N.D.	N.D.	1200	N.D.	N.D.	10 months dead	N.D.
Li	2014	66/male	12	N.D.	Liver first	Right HR	Without CPB, THVE				800	3 h	No	6 months dead	Present, N.D.
Ishino	2015	69/male	12	3	Liver first	Right HR	Without CPB, THVE				1200	6 h 22 min	No	24 months alive	No
Tsang	2017	47/male	19.6	5.8	Liver first	Right HR	CPB	No arrest	N.D.	N.D.	30,000	N.D.	No	7 months alive	Lung

*HCC* hepatocellular carcinoma, *N.D.* not described, *HR* hepatic resection, *CPB* cardiopulmonary bypass, *THVE* total hepatic vascular exclusion

Recently, hepatectomy prior to thrombectomy without CPB has been attempted. There have been five previous reports of cases in which HCC with TT in the right atrium was successfully removed without CPB (Table 1) [3, 7]. According to these reports, no complications or no mortality have been reported. When a TT is excluded from the right atrium after complete mobilization and caudal retraction of the liver, thrombectomy can be done with total hepatic vascular exclusion without CPB. Hepatectomy prior to thrombectomy without CPB was performed in a 77-year-old man with HCC and TT in the right atrium at our institute in 2016. However, a pulmonary embolism due to TT developed in the left pulmonary artery. He died of multiple lung metastases 5 months after surgery. Therefore, hepatectomy prior to thrombectomy carries a risk of sudden cardiac arrest and pulmonary embolism during surgery.

Sugimoto et al. first reported upfront cavo-atrial thrombectomy followed by hepatectomy to prevent sudden death or pulmonary embolism, and there have been no other reports to the best of our knowledge [8]. According to their Japanese report, thrombectomy prior to left hepatectomy was successfully performed without uncontrollable bleeding (blood loss was 3735 mL). In our present case, most bleeding occurred during liver surgery (blood loss was 8200 mL) due to heparinization. Thrombectomy prior to right hepatectomy will prevent uncontrollable bleeding due to inflow obstruction by the ball-valve effect.

For open heart surgery, heparin, cardiac arrest, hypothermia, and cardioplegia under CPB are required. Recently, cardiac surgery has been improved and open heart surgery with beating heart has become possible, removing any limitation of open heart surgery with beating heart. If the TT reaches the right ventricle, thrombectomy will be possible. For liver resection, major hepatectomy such as right or left hepatectomy was required because the size of HCC was huge and the TT also involved the right, middle, or left hepatic vein. In cases of HCC with TT in the right liver, the anterior approach to the IVC or RHV is recommended because this anterior approach is safe for huge HCC in the right liver.

In liver surgery for patients with HCC, safe and oncological management is important. Thrombectomy prior to hepatectomy requires cutting the TT in the IVC. This procedure may induce lung metastasis, and lung metastasis did occur in Sugimoto’s and our patient. However, the rate of lung metastasis is also very high after hepatectomy prior to thrombectomy. Five of 22 cases in previous reports had lung metastasis after hepatectomy prior to thrombectomy. Wakayama et al reported that not only all six patients with TT in the right atrium but also six of seven patients with TT in the IVC had lung metastasis [4]. Furthermore, recent advanced molecular target therapy for lung metastasis is quite effective. Therefore, safe management is more important for patients with HCC and TT in the right atrium.

## Conclusions

This is the first English-language report on cavo-atrial thrombectomy prior to hepatectomy. Cavo-atrial thrombectomy prior to hepatectomy for HCC with TT in the right atrium can be performed safely to prevent sudden cardiac arrest and pulmonary embolism. When a large TT is located in the right atrium and/or is of the pedunculated type, in which case there is a concern for sudden cardiac arrest and pulmonary embolism, cavo-atrial thrombectomy prior to hepatectomy is recommended.

## Abbreviations

AFP: Alpha-fetoprotein
CT: Computed tomography
HCC: Hepatocellular carcinoma
IVC: Inferior vena cava
TT: Tumor thrombus
